# 16S rRNA gene sequencing data of the upper respiratory tract microbiome in the SARS-CoV-2 infected patients

**DOI:** 10.1016/j.dib.2021.107770

**Published:** 2021-12-29

**Authors:** Julia Galeeva, Vladislav Babenko, Ramiz Bakhtyev, Vladimir Baklaushev, Larisa Balykova, Pavel Bashkirov, Julia Bespyatykh, Anna Blagonravova, Daria Boldyreva, Dmitry Fedorov, Ilshat Gafurov, Raushaniya Gaifullina, Elena Galova, Alina Gospodaryk, Elena Ilina, Konstantin Ivanov, Daria Kharlampieva, Polina Khromova, Ksenia Klimina, Konstantin Kolontarev, Nadezhda Kolyshkina, Andrey Koritsky, Vyacheslav Kuropatkin, Vasily Lazarev, Alexander Manolov, Valentin Manuvera, Daria Matyushkina, Maxim Morozov, Ekaterina Moskaleva, Varvara Musarova, Oleg Ogarkov, Elizaveta Orlova, Alexander Pavlenko, Alla Petrova, Natalia Pozhenko, Dmitry Pushkar, Alexander Rumyantsev, Sergey Rumyantsev, Vladimir Rumyantsev, Lyubov Rychkova, Alexander Samoilov, Irina Shirokova, Vyacheslav Sinkov, Svetlana Solovieva, Elizaveta Starikova, Polina Tikhonova, Galina Trifonova, Alexander Troitsky, Alexander Tulichev, Yuri Udalov, Anna Varizhuk, Alexander Vasiliev, Vladimir Veselovsky, Rinat Vereshchagin, Alexey Volnukhin, Gaukhar Yusubalieva, Vadim Govorun

**Affiliations:** a Federal Research and Clinical Center of Physical-Chemical Medicine, Malaya Pirogovskaya, 1a, Moscow 119435, Russian Federation; b Federal Research and Clinical Center of Specialized Medical Care and Medical Technologies, Orekhovy Boulevard, 28, Moscow 115682, Russian Federation; c City Clinical Hospital named after S.I. Spasokukotsky of Moscow Healthcare Department, Academic Consortium, Vuchetich str, 21, Moscow 127206, Russian Federation; d Burnasyan Federal Medical Biophysical Center, Moscow, ul. Marshala Novikova, house 23, Moscow 123098, Russian Federation; e Hospital of the Russian Academy of Sciences, Oktyabrsky prospect, 3, Troitsk 108840, Russian Federation; f Scientific Centre for Family Health and Human Reproduction Problems, 16 Timiryazev str., Irkutsk 664003, Russian Federation; g Privolzhsky Research Medical University, 10/1, Minin and Pozharsky Sq., Nizhny Novgorod 603950, Russian Federation; h Kazan Federal University, 18 Kremlyovskaya str, Kazan 420008, Russian Federation

**Keywords:** SARS-CoV-2, Microbiome, 16S, Upper respiratory tract

## Abstract

The SARS-CoV-2 pandemic is a big challenge for humanity. The COVID-19 severity differs significantly from patient to patient, and it is important to study the factors protecting from severe forms of the disease. Respiratory microbiota may influence the patient's susceptibility to infection and disease severity due to its ability to modulate the immune system response of the host organism.

This data article describes the microbiome dataset from the upper respiratory tract of SARS-CoV-2 positive patients from Russia. This dataset reports the microbial community profile of 335 human nasopharyngeal swabs collected between 2020-05 and 2021-03 during the first and the second epidemic waves. Samples were collected from both inpatients and outpatients in 4 cities of the Russian Federation (Moscow, Kazan, Irkutsk, Nizhny Novgorod) and sequenced using the 16S rRNA gene amplicon sequencing of V3-V4 region. Data contains information about the patient such as age, sex, hospitalization status, percent of damaged lung tissue, oxygen saturation (SpO2), respiratory rate, need for supplemental oxygen, chest computer tomography severity score, SARS-CoV-2 lineage, and also information about smoking and comorbidities.

The amplicon sequencing data were deposited at NCBI SRA as BioProject PRJNA751478.

## Specifications Table

**Table:** 

Subject	Microbiology: microbiome
Specific subject area	Metagenomics
Type of data	Data were presented in FASTA format, Figures}
How the data were acquired	Illumina MiSeq system was used to generate microbiome sequence data
Data format	Raw
Description of data collection	Human nasopharyngeal swabs collected between 2020-05 and 2021-03 during the first and the second epidemic waves. Samples were collected from both inpatients and outpatients in 4 cities of the Russian Federation (Moscow, Kazan, Irkutsk, Nizhny Novgorod)
Data source location	Institution:Federal Research and Clinical Center of Physical-Chemical Medicine City/Town/Region: Moscow Country: Russia
Data accessibility	Repository name: NCBI SRA Data identification number: BioProject PRJNA751478 Direct URL to data: http://www.ncbi.nlm.nih.gov/bioproject/751478

## Value of the Data

• This data is valuable to SARS-CoV-2 and human clinical microbiome researchers interested in understanding the impact of the virus infection on the upper respiratory tract microbial community function and alteration.
• This data will be especially interesting to investigators interested in the association between upper respiratory tract microbiome composition and COVID-19 severity.
• This data will be useful to investigators interested in the association between the host's age, sex, smoking status and the presence of different diseases, and the composition of upper respiratory tract microbiome.

## 1 Data Description

The raw datasets contain 16S rRNA gene sequences produced from nasopharyngeal swab samples collected from SARS-CoV-2 infected patients . The data set possesses 1,552,769 reads with an average of 4,635 reads per sample. Metadata provides the following information about samples: city, hospital id, type of material collected, season of sample collection, technical batch of sequencing and date of sample collection. The patient's state is characterised by the following parameters: age, sex, oxygen saturation (SpO2), respiratory rate, the need for additional oxygen supply (additional O2), chest computer tomography severity score (CT score), percent of affected lung tissue (lung damage) and being hospitalized or on ambulatory treatment (patient status). Information describing the health status of patients and their habits is represented by the following factors: obesity, smoking, smoking in the past (smoking before), diabetes, chronic obstructive pulmonary disease (COPD), inflammatory bowel disease (IBD), arthritis, tuberculosis, hypertension, coronary artery disease, chronic heart failure and asthma, lineage is the SARS-CoV-2 genome Pangolin classification.

**Fig. 1:**
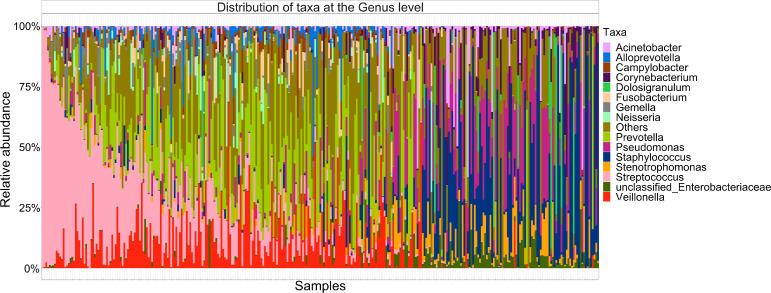
Relative abundance of amplicon sequence variants (% of ASV) describing bacterial community composition of the upper respiratory tract of SARS-CoV-2 positive patients.

Fig. 1 shows the bacterial community composition of the upper respiratory tract of SARS-CoV-2 positive patients.

## 2 Experimental Design, Materials and Methods

The study involved both inpatients and outpatients with COVID-19 who had a confirmed PCR test for the presence of SARS-CoV-2 and signed informed consent to participate in the study. The study did not include patients with cancer.

Samples were collected in 4 cities of the Russian Federation (Moscow, Nizhny Novgorod, Kazan and Irkutsk) with the participation of the following medical centers: Kazan Federal University, Federal State Budgetary Educational Institution of Higher Education «Privolzhsky Research Medical University of the Ministry of Health of the Russian Federation, Federal State Public Scientific Institution «Scientific Centre for Family Health and Human Reproduction Problems», Federal State Budgetary Institution of Healthcare Hospital of the Russian Academy of Sciences (Troitsk), Burnasyan Federal Medical Biophysical Center of Federal Medical Biological Agency, City Clinical Hospital named after S.I. Spasokukotsky of Moscow Healthcare Department, Federal Research and Clinical Center of Specialized Medical Care and Medical Technologies, Federal Biomedical Agency of the Russian Federation.

Nasopharyngeal swabs were recovered from all patients using a dry rayon swab. Collected samples were stored at -70 degrees Celsius. In inpatients, swabs were collected on the day of admission, and in outpatients, on the day of the first visit to the doctor.

Based on the questionnaires, we collected the following information on comorbidities and habits: the presence of obesity, inflammatory bowel disease, diabetes, hypertension, cirrhosis, coronary artery disease, chronic heart failure, asthma, smoking and smoking in the past.

16S rRNA microbiome sequencing analysis:

Nasopharyngeal swabs were proceeded using QIAamp Viral RNA Mini Kit (250) (Qiagen) according to the manufacturer's protocol. 16S library preparation and sequencing were done according to Illumina protocol (16S Metagenomic Sequencing Library Preparation).
We amplified the V3-V4 region of 16S rRNA using primers 16S-F TCGTCGGCAGCGTCAGATGTGTATAAGAGACAGCCTACGGGNGGCWGCAG and 16S-R GTCTCGTGGGCTCGGAGATGTGTATAAGATACAGGATTAACHG . For barcoding the samples, primers were used Nextera XT Index kit v2. The quality of the libraries was assessed using Agilent Bioanalyzer 2100.
In the next step individual amplicons were PCR – indexed and pooled. DNA libraries were sequenced on a MiSeq instrument (Illumina, San Diego, CA, USA) using Miseq reagent kit v3 (Illumina, San Diego, CA, USA).

16s rRNA data processing:

Leftover adapters were removed using Trimmomatic [1] and quality filtering of reads was performed with filter\_and\_trim function from DADA2 package [2]. Then, reads were merged with vsearch [3] and denoising was carried out with deblur software [4].

## Ethics Statement

The study was approved by the ethical committee of RCPCM. All patients gave written informed consent for sample collection and personal data processing. Protocol number № 2020/07.

## CRediT authorship contribution statement

**Julia Galeeva:** Formal analysis, Writing – review & editing, Visualization. **Vladislav Babenko:** Investigation, Methodology. **Ramiz Bakhtyev:** Resources, Data curation. **Vladimir Baklaushev:** Resources, Data curation. **Larisa Balykova:** Resources, Methodology. **Pavel Bashkirov:** Resources, Conceptualization. **Julia Bespyatykh:** Resources, Conceptualization. **Anna Blagonravova:** Resources, Conceptualization. **Daria Boldyreva:** Resources, Conceptualization. **Dmitry Fedorov:** Formal analysis, Investigation, Data curation, Visualization. **Ilshat Gafurov:** Resources, Methodology. **Raushaniya Gaifullina:** Resources, Data curation. **Elena Galova:** Resources. **Alina Gospodaryk:** Resources, Methodology. **Elena Ilina:** Conceptualization, Methodology, Project administration, Writing – review & editing. **Konstantin Ivanov:** Resources, Conceptualization. **Daria Kharlampieva:** Resources, Data curation. **Polina Khromova:** Resources, Conceptualization. **Ksenia Klimina:** Investigation, Methodology. **Konstantin Kolontarev:** Resources, Conceptualization. **Nadezhda Kolyshkina:** Resources, Methodology. **Andrey Koritsky:** Resources, Data curation. **Vyacheslav Kuropatkin:** Resources, Data curation. **Vasily Lazarev:** Resources, Methodology. **Alexander Manolov:** Formal analysis, Investigation, Writing – review & editing, Data curation. **Valentin Manuvera:** Resources, Data curation. **Daria Matyushkina:** Resources, Data curation. **Maxim Morozov:** Investigation. **Ekaterina Moskaleva:** Resources. **Varvara Musarova:** Resources, Methodology. **Oleg Ogarkov:** Resources, Methodology. **Elizaveta Orlova:** Resources, Conceptualization. **Alexander Pavlenko:** Conceptualization, Methodology, Supervision. **Alla Petrova:** Resources, Methodology. **Natalia Pozhenko:** Resources, Conceptualization. **Dmitry Pushkar:** Resources, Conceptualization. **Alexander Rumyantsev:** Resources, Data curation. **Sergey Rumyantsev:** Resources, Data curation. **Vladimir Rumyantsev:** Resources, Data curation. **Lyubov Rychkova:** Resources, Data curation. **Alexander Samoilov:** Resources, Methodology. **Irina Shirokova:** Resources, Conceptualization. **Vyacheslav Sinkov:** Resources, Data curation. **Svetlana Solovieva:** Resources, Data curation. **Elizaveta Starikova:** Writing – review & editing. **Polina Tikhonova:** Formal analysis. **Galina Trifonova:** Resources, Methodology. **Alexander Troitsky:** Resources, Methodology. **Alexander Tulichev:** Resources, Conceptualization. **Yuri Udalov:** Resources, Methodology. **Anna Varizhuk:** Resources, Conceptualization. **Alexander Vasiliev:** Resources, Data curation. **Vladimir Veselovsky:** Investigation, Methodology. **Rinat Vereshchagin:** Formal analysis. **Alexey Volnukhin:** Resources, Conceptualization. **Gaukhar Yusubalieva:** Resources, Data curation. **Vadim Govorun:** Project administration.

## Declaration of Competing Interest

The authors declare that they have no known competing financial interests or personal relationships that could have appeared to influence the work reported in this paper.
